# CallSim: Evaluation of Base Calls Using Sequencing Simulation

**DOI:** 10.5402/2012/371718

**Published:** 2012-12-12

**Authors:** Jarrett D. Morrow, Brandon W. Higgs

**Affiliations:** Center for Biotechnology Education, Johns Hopkins University, Baltimore, MD 21218, USA

## Abstract

Accurate base calls generated from sequencing data are required for downstream biological interpretation, particularly in the case of rare variants. CallSim is a software application that provides evidence for the validity of base calls believed to be sequencing errors and it is applicable to Ion Torrent and 454 data. The algorithm processes a single read using a Monte Carlo approach to sequencing simulation, not dependent upon information from any other read in the data set. Three examples from general read correction, as well as from error-or-variant classification, demonstrate its effectiveness for a robust low-volume read processing base corrector. Specifically, correction of errors in Ion Torrent reads from a study involving mutations in multidrug resistant *Staphylococcus aureus* illustrates an ability to classify an erroneous homopolymer call. In addition, support for a rare variant in 454 data for a mixed viral population demonstrates “base rescue” capabilities. CallSim provides evidence regarding the validity of base calls in sequences produced by 454 or Ion Torrent systems and is intended for hands-on downstream processing analysis. These downstream efforts, although time consuming, are necessary steps for accurate identification of rare variants.

## 1. Introduction

**Figure 1:**
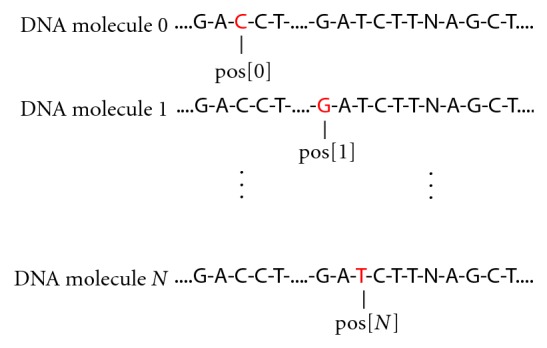
Illustration of the simulated DNA molecules and the polymerase position. Only a single strand of the molecule is represented by the read sequence. The total number of molecules modeled is N, and this would be a snapshot at a later flow, where the polymerase has progressed and dephasing is visible (demonstrated by pos[0] being at a different base than pos[1]). An array pos stores the position of the polymerase associated with each of the N molecules, and its values are initialized to the beginning of the read sequence.

Accurate base calling in high throughput DNA sequencing can be a very challenging task [1, 2], where errors of either biological or technical origin can be introduced. Methods for post-processing of the read data can help mitigate some of this, though various error types can remain in the output result. The sequencing technologies that involve sequential flows of each nucleotide are of interest here and in particular the Roche 454 and Ion Torrent systems. For 454, a pyrosequencing [3] approach is used, while Ion Torrent technology detects changes in pH during base incorporation [4]. One well known source of error in these systems is incomplete extension [5]. That is, a single base or a base within a homopolymeric region might not be incorporated during a flow, and instead, is added during the next flow of the like nucleotide. This dephasing phenomenon, illustrated in Figure 1, accumulates as the number of flows increases, and perturbs the experimental/measured signal in the flowgram. An incorrect base or insertion/deletion (indel) call occurs when this signal perturbation is sufficient to cause an incorrect determination of the number of bases incorporated in the DNA molecule during a flow.

When attempting to detect rare variants or somatic mutations, particularly in the case of mixed samples from tumor tissues [6–8] or rapidly evolving viral strains within a patient [9], read errors may provide false positives, because both rare variants and errors typically present themselves at low frequencies. One difference between read errors and rare variants is that read errors occur at random loci, while rare variants are present at a specific locus. Strategies to identify “real” variants typically depend on quality, depth, coverage, and sequencing technology error rate. However, frequencies of rare variants can often present at <10%, and even lower than 1% in mixed cell populations. Several techniques to correct errors have been developed [10–15], and many of these algorithms either rely on base calls from other reads, or extract statistical or systematic information from other reads. One particularly interesting approach [15] modeled the signal levels at homopolymeric regions across multiple reads by clustering flowgrams, in an effort to determine the probability that the signal is correct, and thereby identify noise in the sequencing process. The CallSim algorithm stands in contrast to these techniques because only the read that contains the base in question is required for CallSim analysis.

In the case of rare variant detection, it is best to avoid discarding any potentially relevant information during the process of error correction, and therefore, retain as much evidence as possible for verification of a rare variant call. CallSim provides evidence to support the validity of such variants and is applicable to both 454 and Ion Torrent PGM data. This algorithm is a robust base call/correcting tool for downstream analyses, complete with a graphical interface to the base calls and signals, and provides either a final variant-or-error classification or “base rescue” mechanism. CallSim is not intended for large-scale base calling; rather, it provides a final classification or rescue of a base/indel in reads, where putative variants have been identified via typical SNP/indel workflows—a very important utility for having confidence in identification of rare variants. It should be noted that terms “read” and “spot” are used interchangeably in the text; however, the spot, as traditionally defined, includes all reads (technical, biological, etc.). In most cases a biological read will be selected as the sequence within the spot that is to be adjusted by CallSim; however the entire spot sequence is simulated because the experimental/measured signal values are for all bases in the spot.

## 2. Materials and Methods

### 2.1. Algorithm

**Figure 2:**
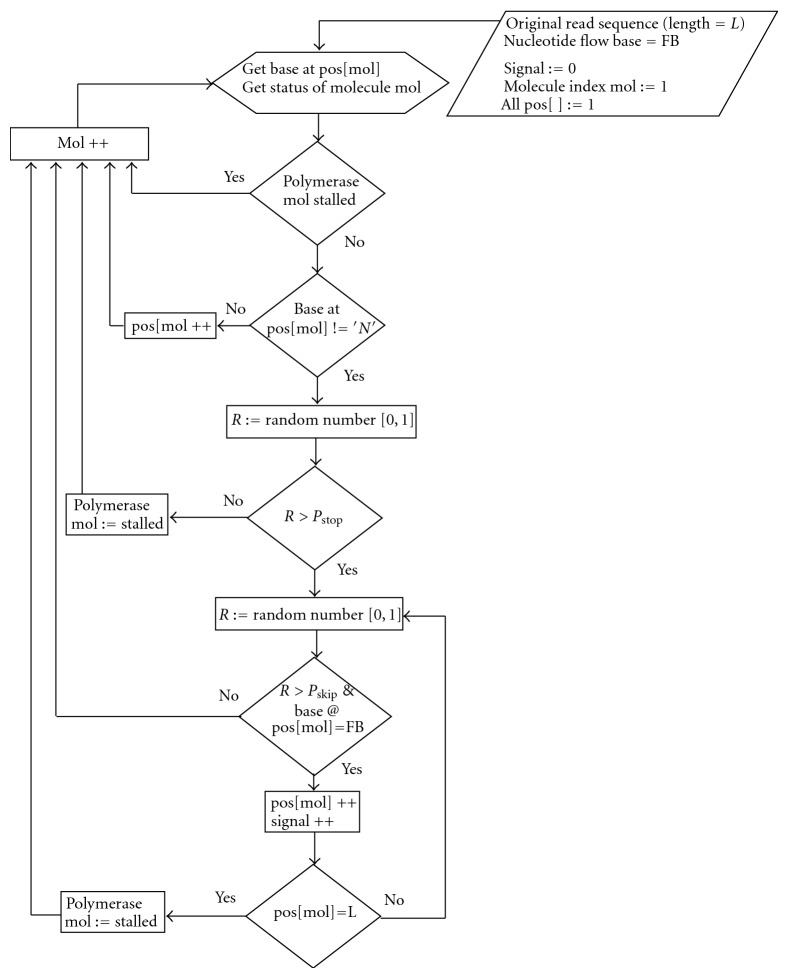
Flowchart of the sequencing simulation process.

The algorithm implemented in CallSim involves the simulation of the sequencing process, by accounting for the random nature of the polymerase on the DNA molecules associated with a single sequencing well or bead, using a Monte Carlo approach [18]. An array variable stores the position of the polymerase associated with each of the N molecules, that each have the sequence of the original read, and the values of the array are initialized to the beginning of that read sequence; a graphical representation of this is provided in Figure 1. Using random number generation and model parameters that are probabilities of particular polymerase activity, the polymerase status and signal-value contributions are determined for each flow. These model parameters and their descriptions are provided in Table 1. In addition, the pseudocode for this simulation process for a single flow is presented in Algorithm 1. As can be seen from this code, Pskip is the probability of the polymerase progressing. The polymerase associated with a particular simulated DNA molecule X will progress and contribute to the signal value for the flow as long as: (1) a generated random number between 0 and 1 is greater than Pskip, (2) the current base (base at the read location stored in pos[X]) matches the flow base, and (3) the end of the molecule is not reached. In order to provide more clarity, a flowchart of the simulation process is also provided (Figure 2). All simulated molecules are examined for the flow, and therefore each will have an opportunity to contribute to the cumulative signal value. These pseudocode operations are repeated for each flow in the experimental/measured data, and the same procedure is used during all simulations, including those in the gradient-descent parameter optimization process.

After these operations are complete, drift effects are included by adding the value (drift∗100∗flow) to the cumulative simulated signal value for the flow, if the user has enabled the optional drift feature. Drift is included in order to account for system effects that tend to increase the experimental/measured signal levels as the number of flows increase. In the case of Ion Torrent systems, it could be a drift in the pH level in the well. This drift effect can be relatively significant for the signals near zero, because those signal values are produced during flows where an in-phase base is not added. The larger signal levels associated with one or more base incorporations can also be affected; however, these values may also be independently altered/lowered by the presence of a non-zero polymerase stall parameter (*P*_stop_ > 0). That is, a non-zero stall parameter means that some polymerase enzymes will become nonfunctional during sequential flows, and hence, the higher signal levels at later flows will be degraded because of the resulting loss of signal contributions.

**Figure 3:**
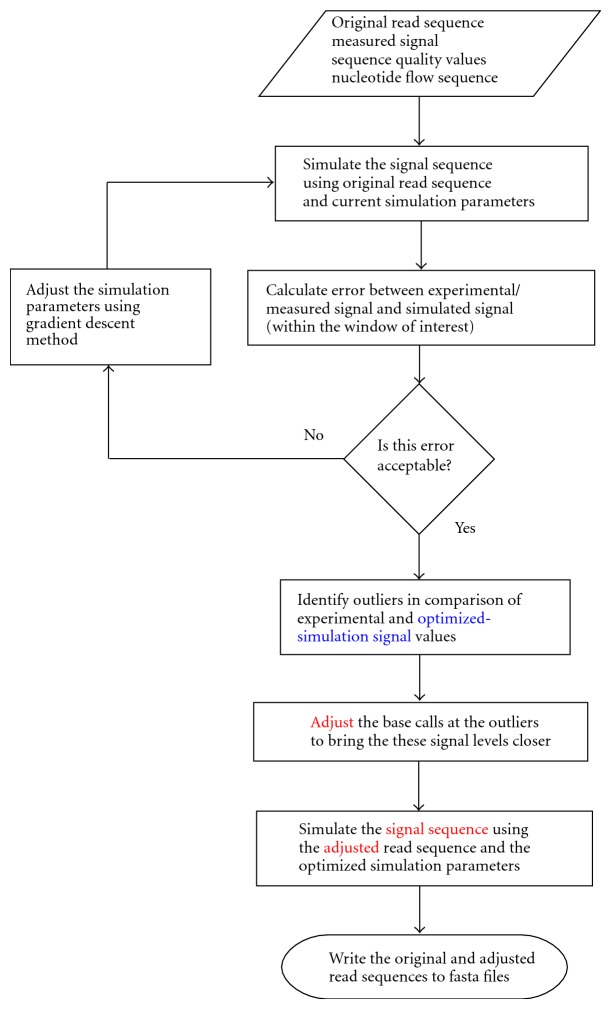
Flowchart of the top-level algorithm. The blue text corresponds to the blue trace in the CallSim Flow Signal Plot window, and the red text corresponds to the red trace in that window.

Given this approach to the simulation of the signals for each flow in the experimental/measured signal data, the high-level view of the algorithm is provided below and is illustrated in a flowchart (Figure 3).

1. Optimize—the parameters of the model to minimize the root mean square (RMS) error between the simulated and experimental/measured signal values over a user-specified window.
2. Find Potential Errors—by comparing the experimental/measured signal values with the simulated signal values (produced using the optimized parameters), and identifying outliers (base calls more likely to be incorrect).
3. Correct—adjust the sequence to compensate for signal discrepancies at outliers, and simulate this adjusted sequence. Write original and adjusted sequences to FASTA files.
4. Evaluate—user evaluates the validity of the correction by observing the signals.

The RMS error is used so that the error levels remain consistent when the user modifies the window size. An absolute error value would be small when the window is small, and larger when all flows are being considered. Lastly, a threshold on the maximum quality value for adjustment may be chosen. That is, if a base has a quality value above this threshold, it will not be adjusted, regardless of other factors.

Adjustments to the optimization and simulation settings are required to achieve good results during the optimization process. For example, the flow window size will affect the ability of CallSim to optimize the simulation parameters, such that the simulation signal values approach the experimental/measured signal values. The initial values for the simulation parameters can also affect the quality of the results, and these are determined by the user. In addition, the gradient descent algorithm, that is used to minimize the RMS error by adjusting the simulation parameters, has a convergence rate parameter that can be modified to “tune” the performance of the optimization. Because of these process variables, each read typically requires unique considerations, and this is a significant reason why this algorithm has not been applied to larger batches of reads at this time.

Because the CallSim algorithm is not applied to batches of reads, it is difficult to accumulate large amounts of data for comparison with other correction and base-calling methods. Therefore, based on the read-by-read analysis presented here, true negatives would be the simulated signal values over all flows that support the correct base calls, and likewise, true positives would be the simulated signal values supporting adjustments that produce correct base calls. A false negative is when an adjustment is not made to produce a correct base call, and a false positive would result when an adjustment produces an incorrect base call. For the validation and test cases presented here, the flow signal plots demonstrate simulation values that track the experimental/measured values well, and hence we have found that false positives are relatively rare when the user has modified the parameters to produce a quality optimization; however false negatives can be more common because an incorrect call is typically associated with a more “noisy” signal region. Although these rates are typically important, it should be noted that CallSim is intended to provide additional evidence for base calls that are likely lacking significant evidence of validity along with having a low frequency, and therefore, in cases where CallSim provides weak support for a adjustment the user may elect to ignore the information instead of accepting a false result. Filtering CallSim information in this manner would be equivalent to discarding base calls with low quality scores.

### 2.2. Software Implementation

CallSim imports information from a read file in text format. This file is produced by extracting data from an SRA format archive using the vdb-dump utility in the SRA Toolkit [19]. An example of a record with the required information is provided in the Supplementary Material. The approach implemented to handle the potentially large text-based read files requires the capabilities of a Linux environment for execution, specifically calls to grep, head, and tail using the Linux shell. CallSim was developed in Java using Netbeans 7.1 and it requires the Java Runtime Environment. It has been evaluated using JRE version 1.6.0_26 on both 64-bit Ubuntu 11.04 and 64-bit CentOS 6.2. The plots are rendered using the JFreeChart library [20], and the required jar files are included in the software distribution.

## 3. Results and Discussion

**Figure 4:**
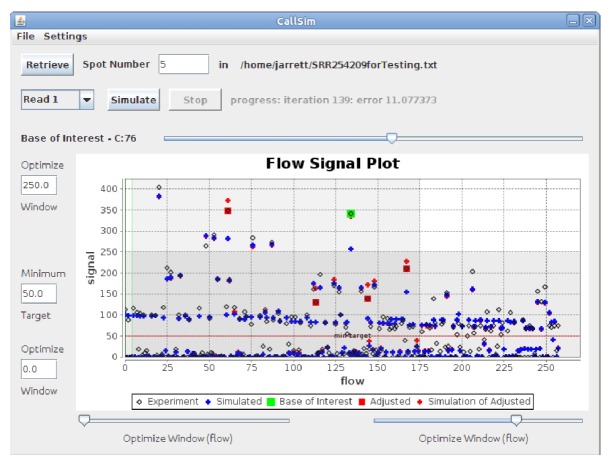
Screenshot of the results for the Ion Torrent validation case. The flow value is the sequencing flow for which the signal was measured (experiment) or simulated. The darker horizontal and vertical regions in these flow signal plots represent the signal-value and flow-number windows, respectively. These regions enclose the experimental/measured signal values that were included in the optimization process. In addition, the green vertical line(s) in the Flow Signal Plot window delineate the signal regions associated with each of the reads within the spot (technical, biological, etc.). In order to provide more clarity on the user interface, a demonstration is provided in the Supplementary Material, available online ay doi:10.5402/2012/371718.

**Figure 5:**
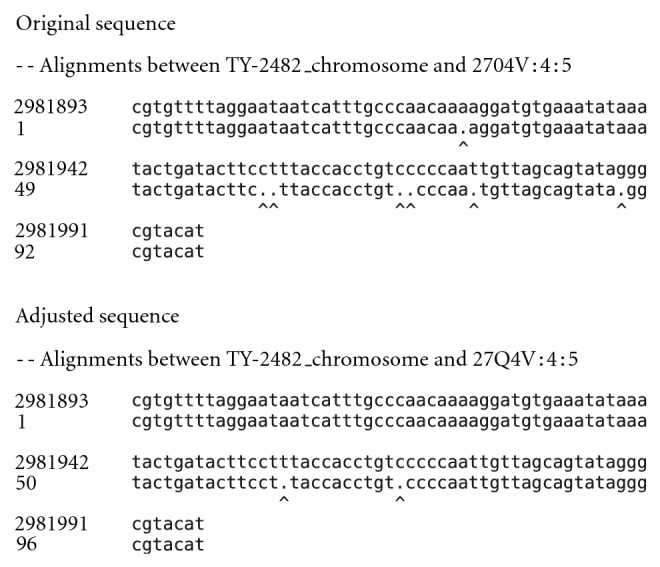
Alignments for the reads from the Ion Torrent validation case to the TY-2482 reference *E. coli* genome.

The algorithm was validated using reads from the *Escherichia coli* outbreak in Germany during the summer of 2011 [21]. Original and adjusted biological reads from both 454 and Ion Torrent sets were mapped to a reference genome, and the ability of CallSim to identify errors was demonstrated by a reduction in the number of mismatches. The screenshot of the analysis results for an Ion Torrent case is shown in Figure 4, where spot number 5 from SRR254209 in SRP007080 [22] has been examined. In the Flow Signal Plot window of this screenshot, the flow numbers correspond to the sequencing flows during which the experimental signals were measured. The red **+** markers indicate the simulated signal values after call adjustments, and when one of these markers is close to the experimental/measured signal marker, stronger evidence is provided to classify that call as an error. As seen from this screenshot, five calls were adjusted with one of these highlighted in green. The experimental/measured and adjusted signal values at the green marker are nearly the same, and therefore the markers are overlayed. The alignment of a subset of the original and adjusted reads for this Ion Torrent validation case is provided in Figure 5, where the alignment was performed using MUMmer 3 [23] with an Ion Torrent + Illumina hybrid assembly (NCBI version) TY-2482 as the reference [24, 25]. From this alignment it can be seen that five mismatches have been eliminated by the five adjustments, and hence, there are five true positives and two false negatives because two mismatches still remain. In addition, the remaining calls are true negatives. Lastly, the quality scores of the five corrected base calls are: A(11):A(11):A(3) adjusted to A:A:A:A, C(14) adjusted to C:C, C(10):C(12):C(4) adjusted to C:C:C:C, T(8) adjusted to T:T, and G(15):G(8) adjusted to G:G:G.

**Figure 6:**
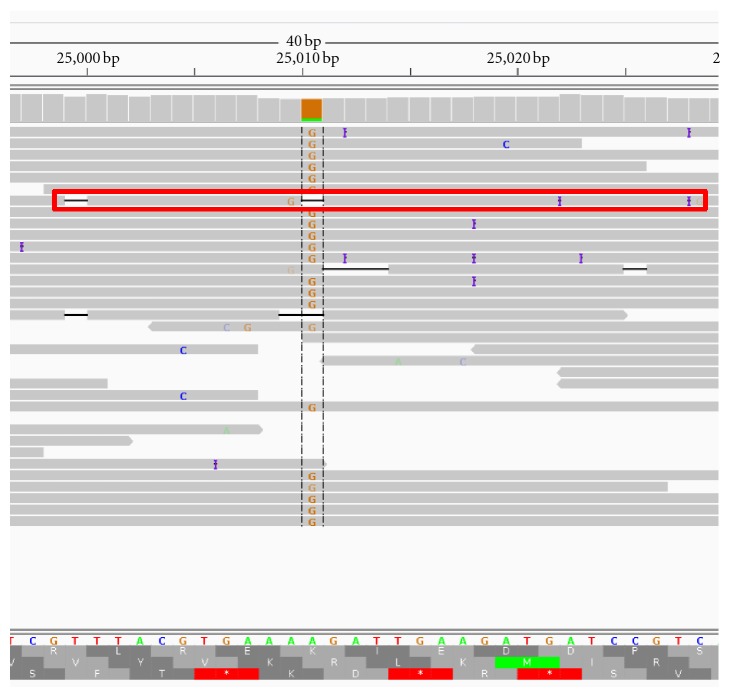
IGV Screenshot of the mapping for the Ion Torrent test case. The mapping of the *Staphylococcus aureus* reads for strain TPS3190 at the locus of interest.

The performance testing included the analysis of data from a study focused on the detection of mutations in multidrug resistant *Staphylococcus aureus* [16]. The Ion Torrent reads for strain TPS3190 (SRR329500 and SRR329501 from SRP007756) were mapped to the *S. aureus* genome JKD6008 using bowtie2-2.0.0-beta5 [26], and the sorted/indexed bam file was viewed using the Integrative Genomics Viewer [27]. A locus of interest identified by the investigators was an expected walR mutation (A->G) at position 25,010 and this region is shown in the IGV screenshot in Figure 6. The first read containing a variant at that location (base other than A or G), was examined (spot number 96038 in SRR329500), and this read is outlined in a red box in Figure 6. The homopolymer AAA should precede 25,010, based on the reference genome and other reads; however the sequence AA is present in this read. The quality scores associated with these bases are A(7):A(7).

**Figure 7:**
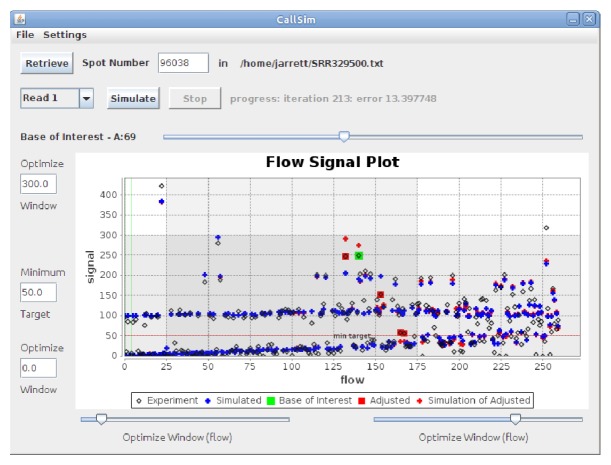
Screenshot of the results for the Ion Torrent test case. Analysis of spot number 96038 in SRR329500 from a *Staphylococcus aureus* study [16].

A screenshot of the analysis results for this Ion Torrent data is provided in Figure 7. In the Flow Signal Plot window of this screenshot, the flow values are the sequencing flows for which the signal was measured (experiment) or simulated, and specifically, the measured flow signal highlighted in green is associated with the aforementioned AA call. The red **+ **marker above it indicates the simulated signal value after the call was adjusted to AAA, and because it is close to the green experimental/measured signal, this is evidence to classify the AA homopolymer call as an error, and illustrates the ability of CallSim to identify errors. Note that the adjustments can result in subtle differences between Simulated and Simulation of Adjusted values in downstream flows because some extensions are carried forward. A subset of the original read and the adjusted read for this Ion Torrent test case is provided in Table 2, where the green bases are the calls associated with the experimental/measured signal highlighted by the green marker in the screenshot of Figure 7. Gaps have been added to these sequences in order to help illustrate the adjusted calls.

**Figure 8:**
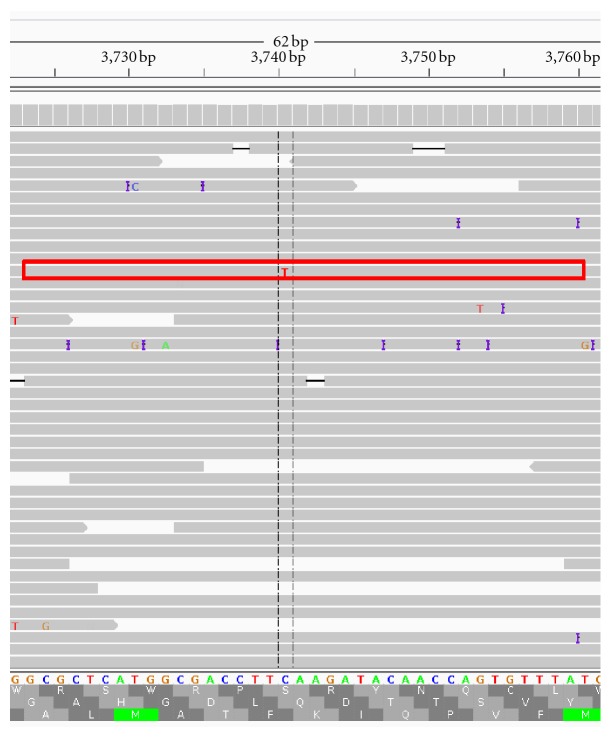
IGV Screenshot of the mapping for the 454 test case The mapping of the West Nile Virus reads SRR331093 at the locus of interest.

CallSim performance testing also included the analysis of data from a study focused on rare variants in mixed viral populations [17]. The 454 reads obtained from a West Nile Virus sample run (SRR331093 from SRP007836) were mapped to the genome consensus assembly JN819311 (Broad Institute project name V5038) using bowtie2-2.0.0-beta5, and the sorted/indexed bam file was viewed using the Integrative Genomics Viewer. In the variant call table provided by the authors, two variants were listed as errors. A read (spot number 3336 in SRR331093) containing the first variant (3774C C->T) was analyzed. This region is shown in the IGV screenshot in Figure 8, where the read of interest is outlined in a red box.

**Figure 9:**
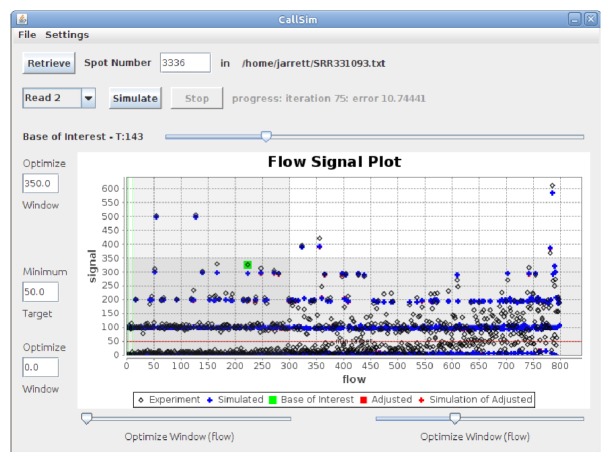
Screenshot of the results for the 454 test case. Analysis of spot number 3336 in SRR331093 from a West Nile Virus study [17].

As can be seen from the Flow Signal Plot window of Figure 9, there is support for preserving the TTT homopolymer call with a Phred quality score of 21. This is due to the fact that the measured (experiment) signal highlighted in green, that corresponds to this call, is close to the simulated value. Because this variant was called an error based on a positive control, this analysis is not a true application example,; however it is a brief illustration of the potential for “read rescue,” where a variant such as 3774C could be retained. As with the previous case, a subset of the original read and the adjusted read for this 454 test case is provided in Table 2, where the green bases are the calls associated with the experimental/measured signal highlighted by the green marker in the screenshot of Figure 9. Additional results and configuration information are available in the Supplementary Material.

## 4. Conclusions

The tool presented here can provide evidence regarding the validity of base calls in sequences produced by Roche 454 or Ion Torrent systems. In the case of rare variants, many error correction techniques that utilize information from other reads have difficulty supporting a low quality base call, because the frequency of rare variants within the population of reads are so low. The algorithm implemented in CallSim does not require information from other reads and therefore may be used as an independent source of evidence to support a error-or-variant determination. 

Intelligent adjustment of the optimization parameters is required to produce acceptable simulation values with respect to experimental/measured values, and therefore, CallSim is intended for hands-on downstream processing efforts with a relatively small quantity of reads. These downstream efforts, although time consuming, are necessary steps for having confidence in identification of rare variants and can provide an alternative to additional sequencing efforts.

## 5. Software Availability and Requirements

 Project name: CallSim.
 Project home page: http://sourceforge.net/projects/callsim/files/.
 Operating system(s): Linux with the Java Runtime Environment installed.
 Programming language: Java.
 Other requirements: JRE 1.6 or higher.
 License: GNU GPL.
 Any restrictions to use by nonacademics: none.

## Supplementary Material

The supplementary material contains additional results and configuration information. In addition, a brief tutorial is included to facilitate the use of CallSim.

## Figures and Tables

**Algorithm 1:**
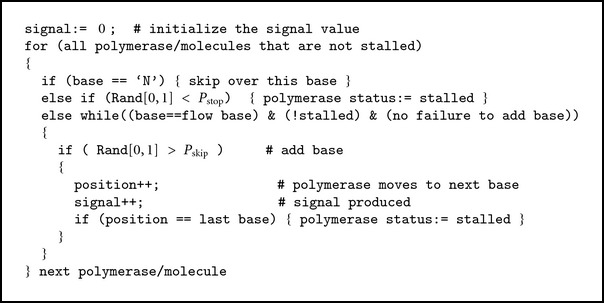
The pseudocode for a single flow in the simulation process. The process is repeated for each flow that was produced during the sequencing process.

**Table 1:** Model parameter descriptions.

Parameter	Description	Comments
*P*_stop_	Prob. of polymerase stall—no incorporation on subsequent flows	Value between 0 and 1
*P*_skip_	Prob. of no base extension—single base or base within a repeat	Value between 0 and 1
Drift	Rate of signal increase over sequential flows	Accounts for process driven signal drift

**Table 2:** Subset of original and adjusted reads.

Test Case	Sequences
(1) Ion Torrent	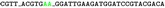
	CGTTTACGTGAAAGGATTGAAGATG_ATCCGT_C_ACA
(2) 454	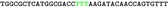
	TGGCGCTCATGGCGACCTTTAAGATACAACCAGTGTTT

These are the reads from the two test cases in the regions of interest, and the bold green bases are the ones corresponding to the green signal of interest.
