# Supplementary material for: CallSim: Evaluation of Base Calls Using Sequencing Simulation
Source: ISRN Bioinform. 2012 Dec 12;2012:371718. doi: 10.5402/2012/371718 (PMC4393072; doi:10.5402/2012/371718)
Supplement: Supplementary file 1 — The supplementary material contains additional results and configuration information. In addition, a brief tutorial is included to facilitate the use of CallSim. [file 371718.f1.pdf]

# CallSim: evaluation of base calls using sequencing simulation

supplementary information

# methods - simulated process

## (polymerase and DNA molecules)

```
mol 0 ....G-A-C-C-T-....-G-A-T-C-T-T-N-A-G-C-T....
               |
             pos[0]

mol 1 ....G-A-C-C-T-....-G-A-T-C-T-T-N-A-G-C-T....
                         |
                       pos[1]
             :                      :
             :                      :
             :                      :
             :                      :

mol N ....G-A-C-C-T-....-G-A-T-C-T-T-N-A-G-C-T....
                             |
                           pos[N]
```

**Illustration of the simulated DNA molecules and the polymerase position.**
In this figure, only a single strand of the molecule is represented by the read sequence. The total number of molecules modeled is N, and this would be a snapshot at a later flow, where the polymerase has progressed and *dephasing* is visible. An array pos[] stores the position of the polymerase associated with each of the N molecules, and its values are initialized to the beginning of the read sequence.

# methods

## (code details)

- CallSim imports information from a read file in text format.  This file is produced by extracting data from an SRA format archive using the vdb-dump utility in the SRA Toolkit.  Please see the example record with the required information in the validation section.

- The approach implemented to handle the potentially large text-based read files requires the capabilities of a Linux environment for execution, specifically calls to grep, head and tail using the Linux shell.

- CallSim was developed in Java using Netbeans 7.1 and it requires the Java Runtime Environment.  It has been evaluated using JRE version 1.6.0_26 on both 64-bit Ubuntu 11.04 and 64-bit CentOS 6.2.

- The plots are rendered using the JFreeChart library, and the required .jar files are included in the distribution.

# settings
## (simulation – main window)

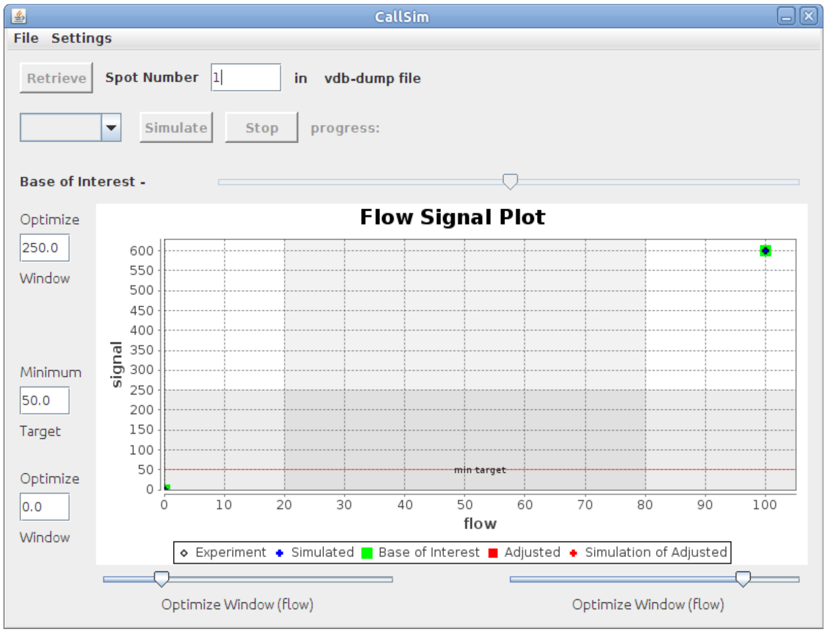


- **signal thresholds** (y-axis light gray region)
  - signal window of measured values included in optimization
- **flow thresholds** (x-axis light gray region)
  - flow window of measured values included in optimization and adjustments
- **min target** (red horizontal line)
  - lowest measured signal that can be adjusted
- **read index**
  - read that is written to fasta file
  - index of the read (0 – technical, 1- biological etc.)

# settings
## (simulation menu)

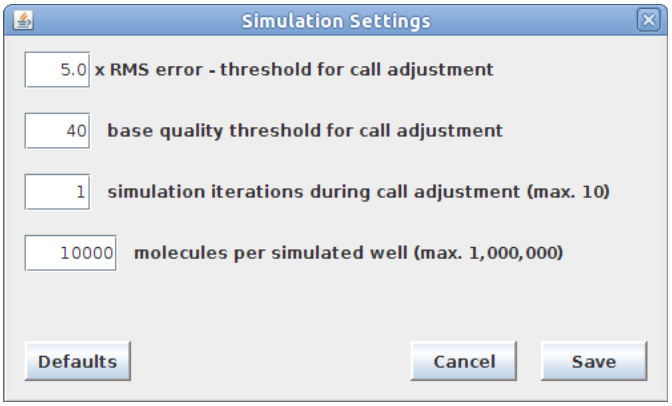


- **cutoff – threshold for call adjustment**
  - illustrated by **blue bar** in later validation plots
  - threshold for difference between measured and simulated signal
  - difference greater than (cutoff * RMS_error) --> correction

- **base quality threshold for call adjustment**
  - maximum base sequencing quality that will permit a base correction

- **simulation iterations during call adjustment**
  - number of iterations performed for each simulation
  - 1 should provide good performance (with sufficient number of molecules)

- **molecules per simulated well/bead**
  - number of DNA molecules simulated (10,000 provides good performance)

# settings
## (optimization menu)

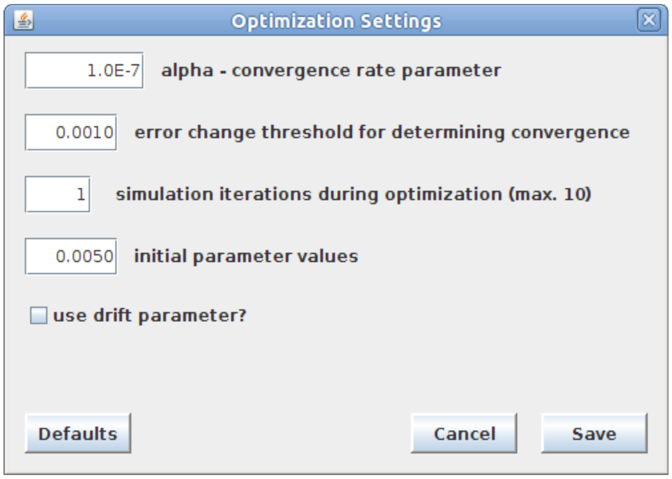


- **alpha - convergence rate**
  - parameter of gradient descent optimization algorithm
  - smaller value provides longer execution with finer resolution

- **error change threshold for determining convergence**
  - automatically halts optimization when iteration error change is below threshold
  - can also halt optimization manually if convergence is not achieved

- **simulation iterations during optimization**
  - number of iterations performed for each simulation during optimization
  - 1 should provide good performance (with sufficient number of molecules)

- **initial parameter values**
  - starting value for parameters to be optimized

- **use drift parameter**
  - select whether to include the signal drift parameter
  - drift help to account for a process driven upward signal drift

# performance validation

The algorithm was validated using reads from the Escherichia coli outbreak in Germany during the summer of 2011, where pathogenic genes were the focus.

Original and adjusted biological reads from both 454 and Ion Torrent sets were mapped to a reference genome  using MUMmer (nucmer).

The ability of CallSim to identify errors was demonstrated by a reduction in the number of mismatched bases.

# sequencing data sources
(for validation)

- **454 NGS data:**
  - SRP009694  Performance comparison of bench-top high-throughput sequencing platforms
  - submitters: “Both the Ion Torrent and 454 Junior suffer from homopolymeric tract miscalls.”
  - Sequencing of E. coli STEC O104:H4 from a recent outbreak in Germany
  - 454 GS Junior
  - http://trace.ncbi.nlm.nih.gov/Traces/sra/sra.cgi?study=SRP009694

- reference genome(s):
  - 06/06/11 Ion Torrent+Illumina hybrid assembly (NCBI version)
    - Escherichia coli TY-2482.contig.20110606.fa.gz
    - provides a genome for determining base-call errors
    - http://gigadb.org/e-coli/
  - Escherichia coli 55989 chromosome, complete genome
    - more appropriate for an assembly reference
    - http://www.ncbi.nlm.nih.gov/nuccore/NC_011748.1

# sequencing data sources
## (for validation)

- **Ion Torrent NGS data**: outbreak last summer in Germany
  - SRP007080  Whole Genome Sequencing of Escherichia coli O104:H4 str. LB226692
    - http://trace.ncbi.nlm.nih.gov/Traces/sra/?study=SRP007080

- reference genomes:
  - 06/06/11 Ion Torrent+Illumina hybrid assembly (NCBI version)
    - Escherichia coli TY-2482.contig.20110606.fa.gz
    - provides a genome for determining base-call errors
    - http://gigadb.org/e-coli/
  - Escherichia coli 55989 chromosome, complete genome
    - more appropriate for an assembly reference
    - http://www.ncbi.nlm.nih.gov/nuccore/NC_011748.1

# methods
## (read information)

- short read archive text file
  - extract using SRA toolkit (vdb-dump)
  - summary of some data obtained for each read:
    - FLOW_CHARS: TACGTACGTCTGAGCATCGATCGAT
      - (order of base flow during sequencing)
    - READ: TCAGGGGTTCAGTCGTTGAGTCCG
    - NREADS: 4
      - (technical and biological)
    - READ_SEG: [0, 4], [4, 128], [132, 44], [176, 86]
      - (coordinates of technical and biological reads)
    - QUALITY: 20, 20, 13, 13, 13, 13, 3, 13, 20, 25, 25, 25

- next three slides contain an example of the specific record information and format required

# spot #5, SRR254209.txt

```
        BASE_COUNT: 177713150
    BIO_BASE_COUNT: 173801266
 CLIP_ADAPTER_LEFT: 0
CLIP_ADAPTER_RIGHT: 100
 CLIP_QUALITY_LEFT: 5
CLIP_QUALITY_RIGHT: 108
    CMP_BASE_COUNT: 177713150
      COLOR_MATRIX: 0, 1, 2, 3, 4, 1, 0, 3, 2, 4, 2, 3, 0, 1, 4, 3, 2, 1, 0, 4, 4, 4, 4, 4, 4
            CSREAD: 0212231110003202033032130013001011002023111200333300331212331202203101102112001
031103231213332033131132110321231013322222321222121301203111
            CS_KEY: TT
         CS_NATIVE: false
    FIXED_SPOT_LEN: 0
        FLOW_CHARS: TACGTACGTCTGAGCATCGATCGATGTACAGCTACGTACGTCTGAGCATCGATCGATGTACAGCTACGTACGTCTGAGC
ATCGATCGATGTACAGCTACGTACGTCTGAGCATCGATCGATGTACAGCTACGTACGTCTGAGCATCGATCGATGTACAGCTACGTACGTCTGAGCATCGATC
GATGTACAGCTACGTACGTCTGAGCATCGATCGATGTACAGCTACGTACGTCTGAGCATCGATCGATGTACAGCTACG
      KEY_SEQUENCE: TCAG
             LABEL:
         LABEL_LEN: 0, 0
         LABEL_SEG: [0, 0], [0, 0]
       LABEL_START: 0, 0
       MAX_SPOT_ID: 977971
       MIN_SPOT_ID: 1
              NAME: 27Q4V:4:5
            NREADS: 2
          PLATFORM: SRA_PLATFORM_454
          POSITION: 1, 3, 6, 8, 10, 12, 17, 19, 21, 21, 21, 21, 24, 26, 26, 28, 28, 33, 34, 34, 37,
39, 45, 49, 49, 49, 51, 54, 54, 54, 56, 56, 61, 62, 62, 62, 63, 63, 66, 69, 72, 73, 76, 77, 77, 77, 81,
84, 85, 88, 88, 88, 89, 92, 93, 97, 100, 102, 105, 109, 111, 113, 113, 114, 117, 117, 120, 125, 125,
126, 128, 128, 129, 132, 133, 135, 135, 135, 141, 141, 145, 147, 149, 149, 152, 154, 157, 158, 159,
161, 162, 165, 166, 168, 168, 170, 172, 177, 180, 182, 184, 185, 189, 190, 192, 192, 196, 198, 199,
201, 205, 207, 207, 208, 209, 212, 215, 216, 218, 220, 223, 224, 225, 228, 230, 232, 237, 239, 241,
243, 246, 246, 248, 250, 250, 253, 254, 256, 258
           QUALITY: 32, 32, 32, 32, 32, 32, 32, 32, 32, 32, 32, 7, 32, 26, 19, 26, 19, 26, 26, 19, 26,
26, 20, 15, 15, 6, 15, 21, 21, 9, 21, 14, 11, 11, 11, 3, 11, 17, 19, 21, 21, 17, 17, 17, 19, 9, 28, 28,
19, 19, 19, 11, 17, 19, 19, 19, 21, 21, 20, 15, 14, 14, 7, 14, 13, 14, 16, 16, 12, 11, 11, 5, 8, 8, 10,
10, 12, 4, 12, 7, 8, 12, 11, 8, 12, 15, 12, 11, 12, 11, 11, 11, 14, 15, 8, 11, 11, 12, 11, 11, 11, 11,
11, 9, 9, 5, 8, 9, 8, 8, 8, 8, 5, 8, 8, 8, 8, 8, 8, 8, 8, 8, 8, 8, 8, 9, 8, 8, 8, 8, 8, 5, 8, 8, 8, 8,
8, 8, 8
              READ: TCAGCGTGTTTTAGGAATAATCATTTGCCCAACAAAGGATGTGAAATATAAATACTGATACTTCTTACCACCTGTCCCAA
TGTTAGCAGTATAGGCGTACATCACCGACTACCATAGAGAGCTGAGACTGCCAGGCACA
         READ_DESC: [seg.start=0, seg.len=4, type=0, cs_key=84, label=], [seg.start=4, seg.len=135,
type=1, cs_key=84, label=]
       READ_FILTER: SRA_READ_FILTER_PASS, SRA_READ_FILTER_PASS
          READ_LEN: 4, 135
          READ_SEG: [0, 4], [4, 135]
        READ_START: 0, 4
         READ_TYPE: SRA_READ_TYPE_TECHNICAL, SRA_READ_TYPE_BIOLOGICAL
            REGION:
            SIGNAL: 112, 0, 89, 0, 0, 116, 0, 106, 18, 103, 4, 101, 10, 14, 0, 0, 100, 1, 99, 4, 404,
0, 6, 118, 0, 212, 2, 202, 0, 0, 19, 0, 100, 193, 0, 1, 100, 0, 88, 3, 0, 7, 0, 0, 116, 5, 9, 0, 264,
6, 91, 9, 7, 290, 0, 184, 0, 3, 0, 0, 119, 348, 184, 1, 0, 117, 0, 0, 91, 0, 6, 107, 97, 0, 0, 78, 283,
0, 3, 0, 98, 1, 26, 113, 85, 0, 19, 274, 88, 0, 0, 104, 86, 0, 3, 0, 79, 30, 0, 95, 14, 95, 0, 16, 82,
0, 0, 0, 101, 0, 74, 0, 161, 130, 8, 9, 196, 19, 0, 83, 24, 9, 0, 0, 171, 77, 8, 155, 101, 0, 8, 70,
55, 6, 341, 9, 15, 8, 0, 0, 157, 6, 20, 7, 138, 19, 73, 8, 166, 16, 5, 68, 14, 96, 0, 0, 92, 65, 71, 0,
68, 73, 0, 0, 79, 86, 40, 210, 10, 71, 0, 72, 3, 25, 0, 0, 61, 4, 0, 78, 0, 69, 16, 138, 81, 33, 0, 13,
78, 83, 23, 153, 25, 0, 0, 71, 0, 53, 80, 24, 75, 11, 0, 49, 75, 13, 204, 56, 73, 0, 18, 75, 0, 41, 65,
108, 0, 62, 0, 75, 6, 26, 112, 54, 82, 0, 0, 62, 0, 105, 1, 82, 3, 0, 0, 0, 89, 0, 67, 0, 74, 0, 67, 8,
0, 157, 0, 132, 0, 166, 0, 0, 75, 70, 0, 75, 14, 75, 11, 33
        SIGNAL_LEN: 260
        SPOT_COUNT: 977971
         SPOT_DESC: spot_len=139, fixed_len=0, signal_len=260, clip_qual_right=100, num_reads=2
        SPOT_GROUP:
           SPOT_ID: 5
          SPOT_LEN: 139
          TRIM_LEN: 96
        TRIM_START: 4
                 X:
                 Y:
```

# getting started
(analysis of validation data)

- **download the CallSim.zip file that is available at**
  - the Bioinformatics website and
  - http://sourceforge.net/p/callsim
- **unzip/extract the files and directory structure in CallSim.zip**
- **execute from within a terminal**
  - move to the CallSim folder 'cd CallSim'
  - make the CallSim.jar file executable 'chmod +x CallSim.jar'
  - **ensure Java Runtime Environment is installed**
  - execute CallSim 'java -jar CallSim.jar'

(to produce the read file in text format)

- **extract data from an SRA format archive**
  - using the vdb-dump utility in the SRA Toolkit
  - from within a terminal use the command template:
    - '*pathToSRAToolkit*/vdb-dump *SRAFile*.sra > *OutputFileName*'

# getting started: step-by-step
## (validation simulation example)

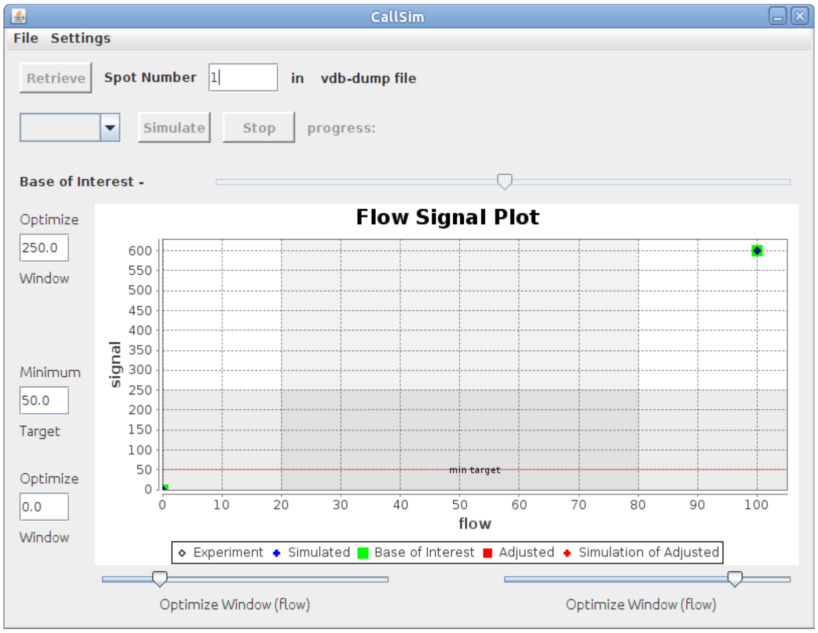


- from the main GUI window, choose the text-format read file
  - 'file' menu -> 'Open vdb-dump file'
  - select the file '**SRR254209forTesting.txt**' and click 'Open' (file was included with code)

- choose the spot of interest in that file
  - place **5** in the 'Spot Number' text box
  - click on 'Retrieve' - measured signal values should appear in the Signal Flow Plot window

- adjust the settings below (leave default values for those not mentioned)
  - 'Settings' menu -> 'Simulation'
    - cutoff = 3.0 (x RMS error)
    - quality threshold = 40
    - simulation iterations = 1
    - number of molecules = 10,000
  - click 'Save' to save the settings

- 'Settings' menu -> 'Optimization'
    - alpha = 1E-8
    - simulation iterations = 1
    - initial parameter values = 0.005
    - no drift – (upward drift not seen in flow signal plot)
- click 'Save' to save the settings

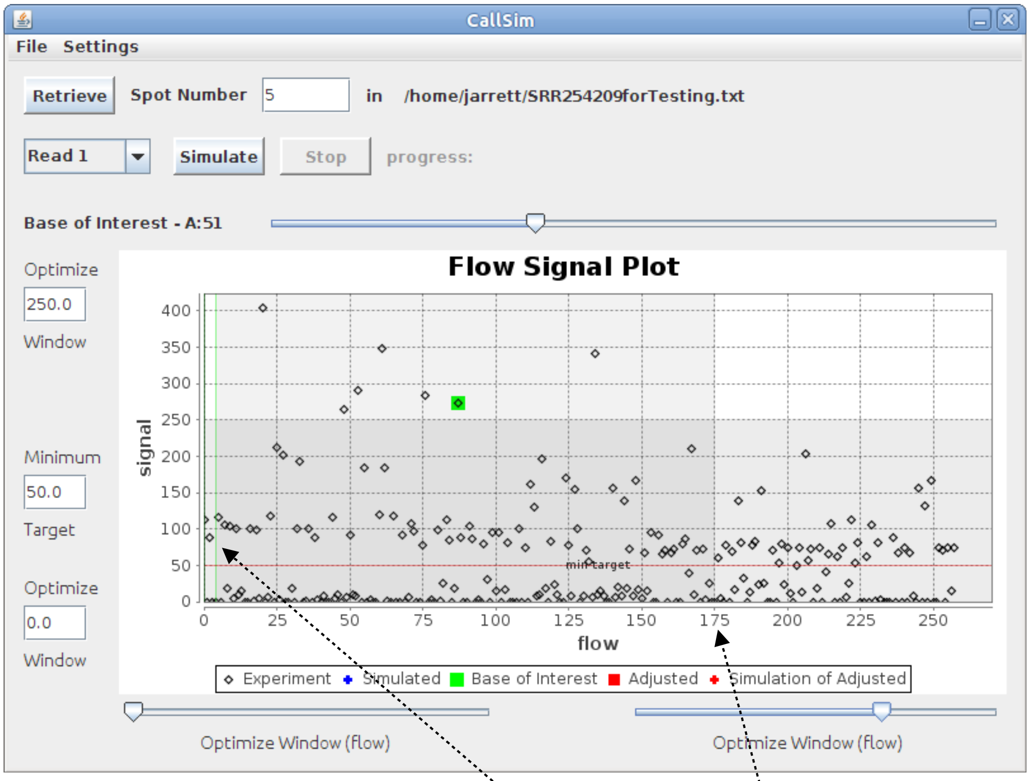


- specify optimization window
    - specify flows included by using the 'Optimize Window (flow)' sliders
        - place the left side of flow window at the green vertical line in the plot
        - place the right side of flow window at approximately 175
    - specify the signal levels included by
        - keeping the default values of 0.0 and 250.0 in the 'Optimize Window' text boxes

- click on 'Simulate'
    - wait for the optimization to converge, based on the settings, or click 'Stop' when needed
        - the original read and the adjusted read are both written to FASTA files

# validation settings

(single read from Ion Torrent)

The error should reach a value of approximately 11, and the results are shown below:

## validation example

(spot #5, SRR254209)

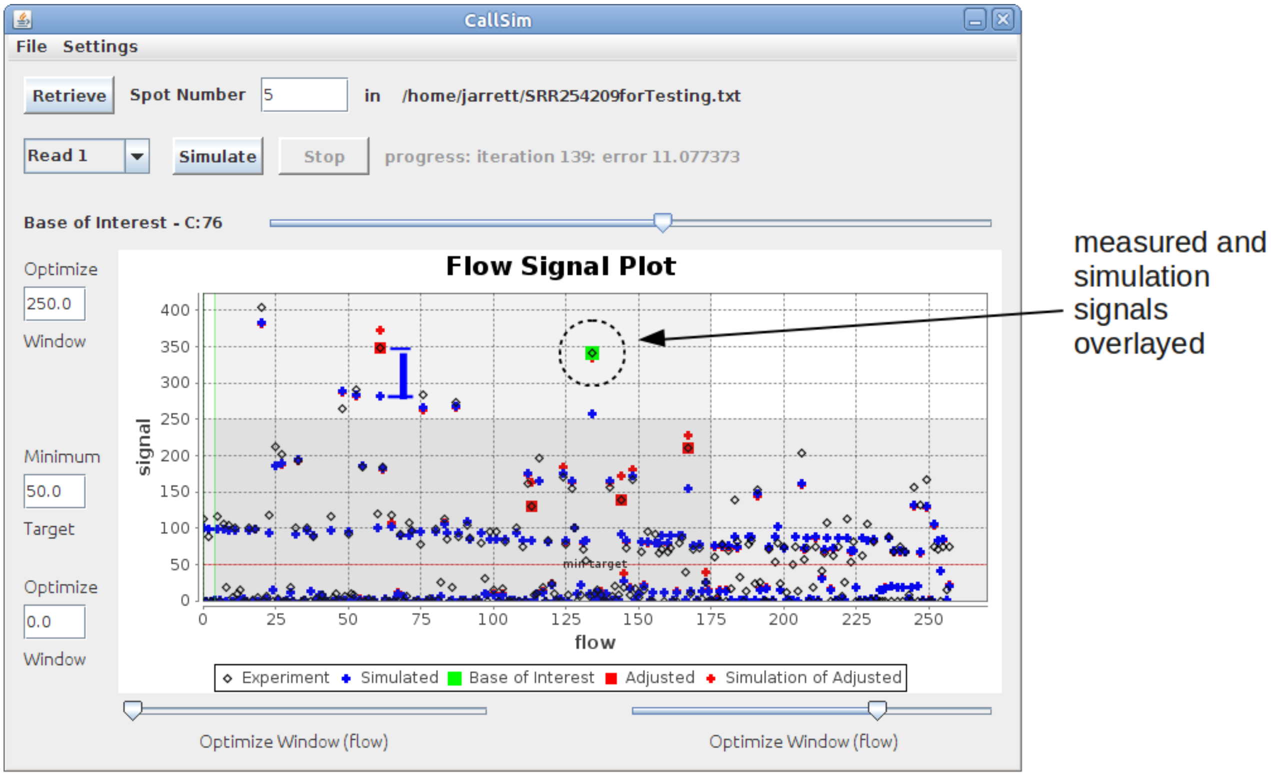

# results – alignment
## (single read from Ion Torrent)

**five** SNPs were eliminated by the four adjustments

**original sequence**

```
-- Alignments between TY-2482_chromosome and 27Q4V:4:5

2981893    cgtgttttaggaataatcatttgcccaacaaaaggatgtgaaatataaa
1          cgtgttttaggaataatcatttgcccaacaa.aggatgtgaaatataaa
                                          ^
2981942    tactgatacttcctttaccacctgtcccccaattgttagcagtataggg
49         tactgatacttc..ttaccacctgt..cccaa.tgttagcagtata.gg
                       ^^          ^^     ^             ^
2981991    cgtacat
92         cgtacat
```

**adjusted sequence**

```
-- Alignments between TY-2482_chromosome and 27Q4V:4:5

2981893    cgtgttttaggaataatcatttgcccaacaaaaggatgtgaaatataaa
1          cgtgttttaggaataatcatttgcccaacaaaaggatgtgaaatataaa

2981942    tactgatacttcctttaccacctgtcccccaattgttagcagtataggg
50         tactgatacttcct.taccacctgt.ccccaattgttagcagtataggg
                         ^          ^
2981991    cgtacat
96         cgtacat
```

# validation settings

(single read from 454)

- cutoff = 4.0 (x RMS error)
- number of molecules = 10,000
- simulation iterations = 1
- quality threshold = 40
- no drift – (upward drift not seen in flow signal plot)
- alpha = 1E-8
- initial parameter values = 0.002

## results

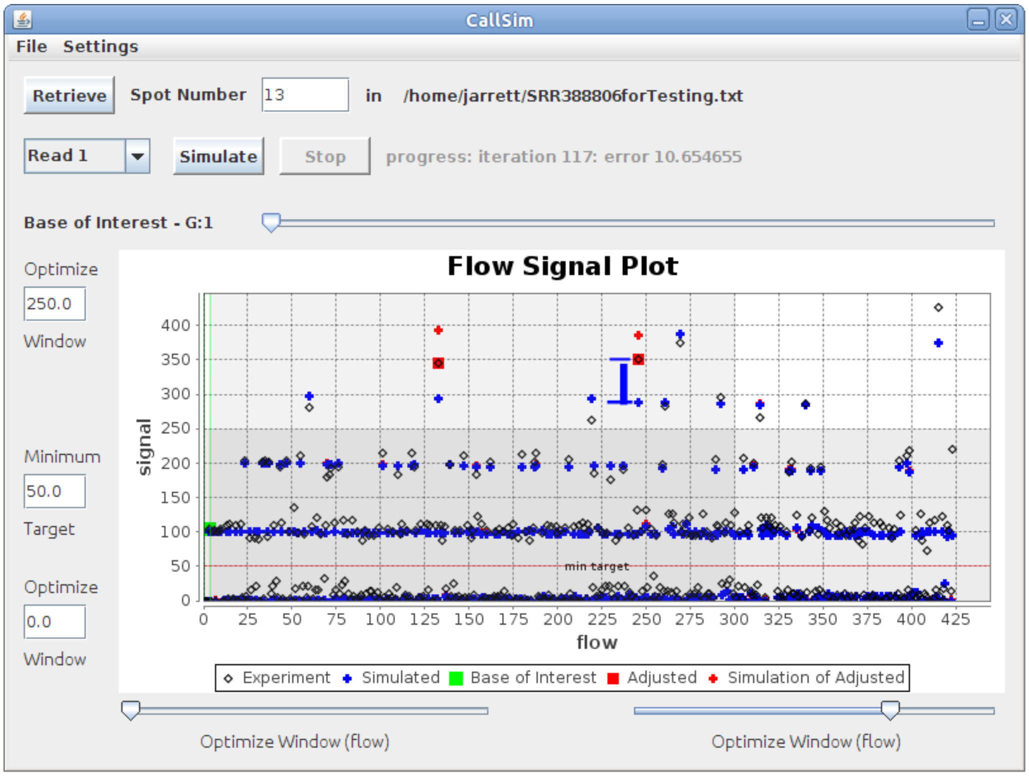

# results – alignment
## (single read from 454)

**two** SNPs were eliminated by the four adjustments

**original sequence**

```
-- Alignments between TY-2482_chromosome and G310ZZ001ACA3X

5203948    acagcaggcagaaggtaatggtggtgaggatttatcaccggttcttgat
1          acagcaggcagaaggtaatggtggtgaggatttatcaccggttcttgat

5203997    gcgatgaatgccgtgccggtgctgaaaacgtggcaggagtccgatccag
50         gcgatgaatgccgtgccggtgctgaa.acgtggcaggagtccgatccag
                                     ^
5204046    atcgcttctcggttgctgtatccatcgacgggaagctccagaatgaccc
98         atcgcttctcggttgctgtatccatcgacgggaagctccagaatgacc.
                                                           ^
5204095    cgcatggaaagacaaaacg.ctcactgaacgtttcgctgaagtggcccg
146        cgcatggaaagacaaaacgactcactgaacgtttcgctgaagtggcccg
                              ^
5204143    tcgtacgcaggttgctttcggtgaagtcagtgagtcgtctgctgacaac
195        tcgtacgcaggttgctttcggtgaagtcagtgagtcgtctgctgacaac

5204192    aaggcagaca
244        aaggcacaca
                 ^
```

**adjusted sequence**

```
-- Alignments between TY-2482_chromosome and G310ZZ001ACA3X

5203948    acagcaggcagaaggtaatggtggtgaggatttatcaccggttcttgat
1          acagcaggcagaaggtaatggtggtgaggatttatcaccggttcttgat

5203997    gcgatgaatgccgtgccggtgctgaaaacgtggcaggagtccgatccag
50         gcgatgaatgccgtgccggtgctgaaaacgtggcaggagtccgatccag

5204046    atcgcttctcggttgctgtatccatcgacgggaagctccagaatgaccc
99         atcgcttctcggttgctgtatccatcgacgggaagctccagaatgaccc

5204095    cgcatggaaagacaaaacg.ctcactgaacgtttcgctgaagtggcccg
148        cgcatggaaagacaaaacgactcactgaacgtttcgctgaagtggcccg
                              ^
5204143    tcgtacgcaggttgctttcggtgaagtcagtgagtcgtctgctgacaac
197        tcgtacgcaggttgctttcggtgaagtcagtgagtcgtctgctgacaac

5204192    aaggcagaca
246        aaggcacaca
                 ^
```

# test case #1
## Ion Torrent

- Detection of mutations in multidrug resistant Staphylococcus aureus
  - Howden et al.
  - reads for strain TPS3190
    - SRP007756 -> SRX090653 -> SRR329500 & SRR329501
    - mapped to S.aureus genome JKD6008 using bowtie2-2.0.0-beta5
    - viewed using IGV in next slide
  - chose the first two reads demonstrating variation from G
    - red box also encloses other variants at other locations in the read
  - analysis of the first read is from the application note

## locus of Interest – base 25010

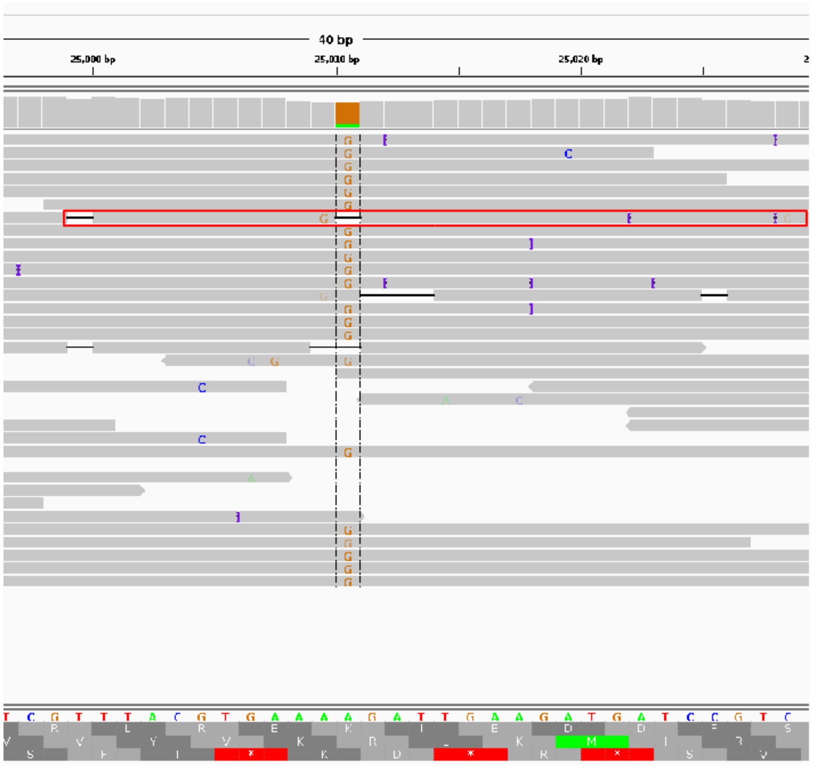


chosen read in red box:
spot # 96038
SRR329500

locus of interest from Howden et al. section - “Generation and Whole Genome Sequencing of walKR Mutants”

# settings

(single read from Ion Torrent)

- cutoff = 3.0 (x RMS error)
- number of molecules = 10,000
- simulation iterations = 1
- quality threshold = 40
- using drift – (upward drift is seen in flow signal plot)
- alpha = 1E-8
- initial parameter values = 0.005

# results

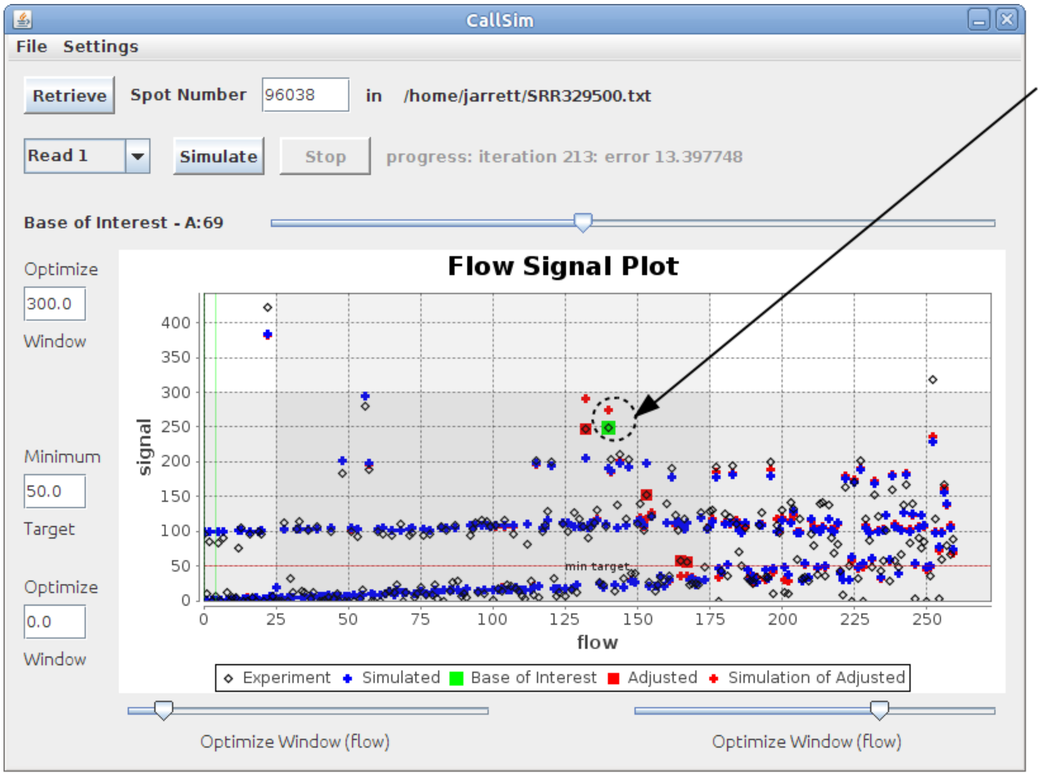


homopolymer
AA -> AAA @A70
so base G variant
becomes aligned

also:

TT -> TTT @T63
eliminates gap

GG -> G @G83
eliminates insert

and

A -> _ @A90
G -> _ @G92
so C91 becomes
aligned

# locus of Interest – base 25010
# (second read)

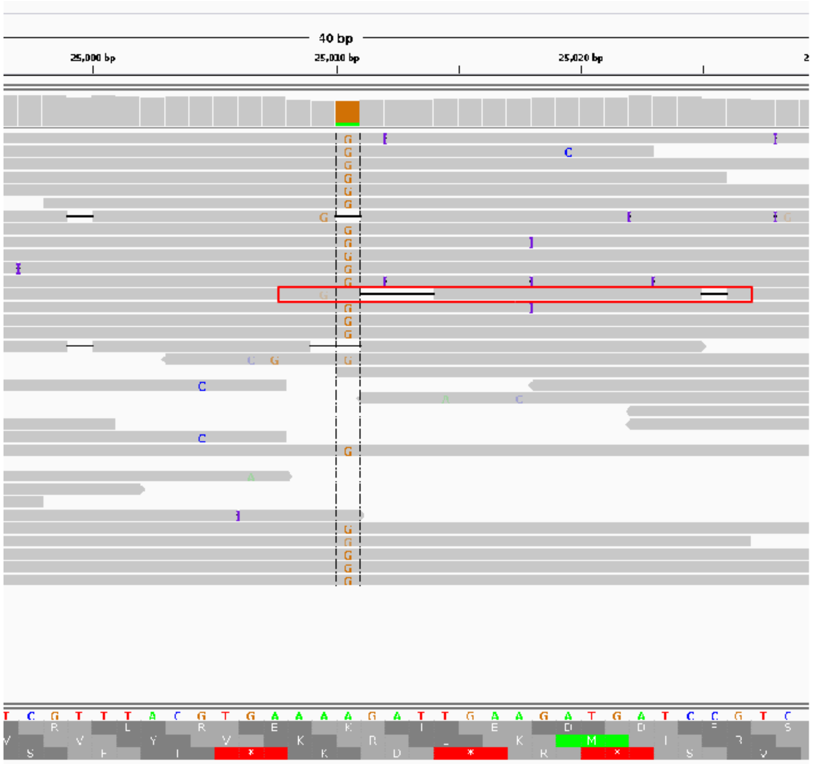


second read in red box:
spot #10375
SRR329501

locus of interest from Howden et al. section - “Generation and Whole Genome Sequencing of walKR Mutants”

# settings

## (single read from Ion Torrent)

- cutoff = 3.0 (x RMS error)
- number of molecules = 10,000
- simulation iterations = 1
- quality threshold = 40
- no drift – (upward drift not seen in flow signal plot)
- alpha = 1E-8
- initial parameter values = 0.005

# results

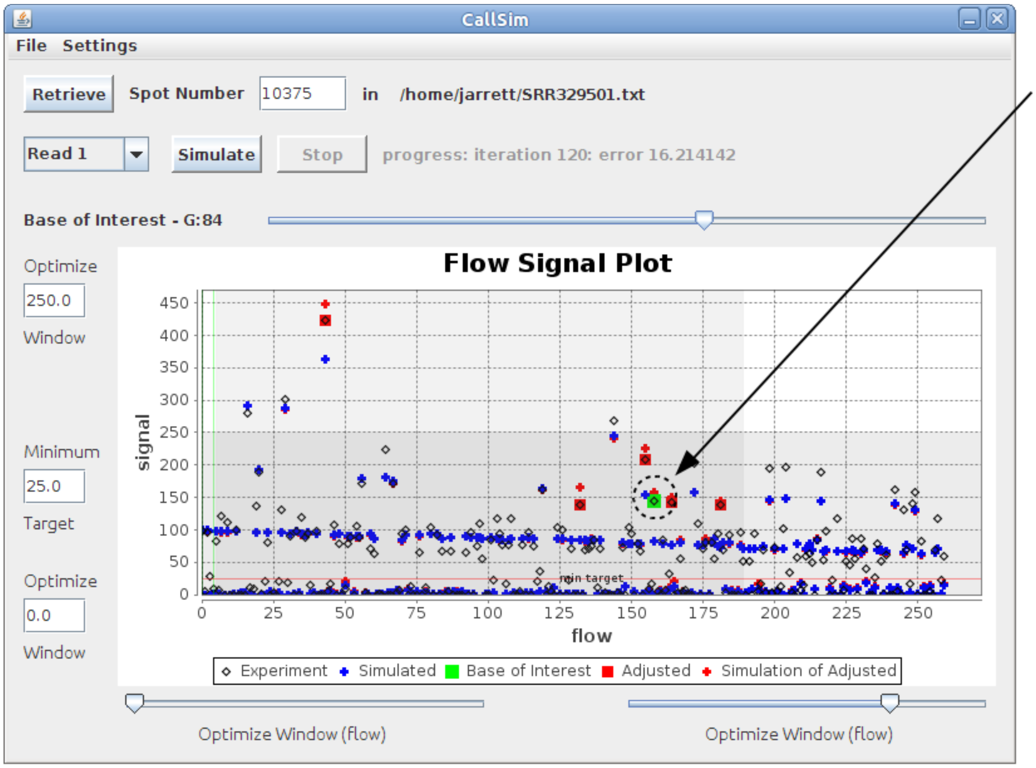


homopolymer
AA -> AAA @A83
G -> GG @G84
so base G variant
becomes aligned

also:
T -> TT @T86
eliminates gap

downstream
C -> CC @C96
eliminates gap

and upstream
T -> TT @T68
eliminates gap

however
4G -> 5G @G27
appears to be
an error

# test case #2

## 454

- Rare Variants in Mixed Viral Populations
  - Macalalad et al.
  - reads for West Nile Virus
    - SRP007836 -> SRX091924 -> BI project name V5038
    - 454 reads: SRR331093
    - mapped to genome assembly JN819311 (10451 bp ss-RNA linear)
      - West Nile virus isolate WNV-1/BID-V5038, complete genome
      - appears to have been assembled from SRR331093 reads
      - using bowtie2-2.0.0-beta5
    - viewed using IGV (later slide)

- chose the first read containing the each rare variant (see next slide)
  - red box also encloses variants at other locations in the read

- analysis of the first read is from the application note

## from supplemental Table S1
## (Macalalad et al.)

| Variant | Str1 | Str2 | Str3 | Str4 | Str5 | Str6 | Str7 | Str8 | Exp. Freq. | Obs. Freq. | V-Phaser |
|---|---|---|---|---|---|---|---|---|---|---|---|
| 3774C | . | . | . | . | . | . | . | T | 0.007 | 0.003 | Error |
| 3625A | . | . | . | . | . | . | . | T | 0.007 | 0.004 | Error |

- these are two locations demonstrating an error
- location in genome assembly JN819311 is offset by 34 bases
  - 3740C – analysis of first read with T variant provided
  - 3591A – analysis of first read with T variant provided

# locus of Interest – base 3740

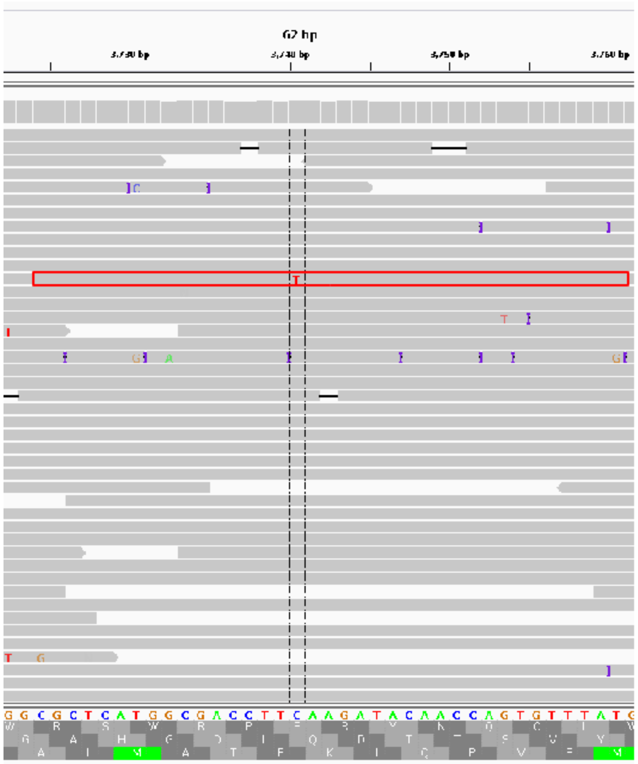

spot # 3336 outlined in red box

locus of interest from Macalalad et al.

rare variant listed as an error

has phred quality score of 21

# read of Interest – extended view

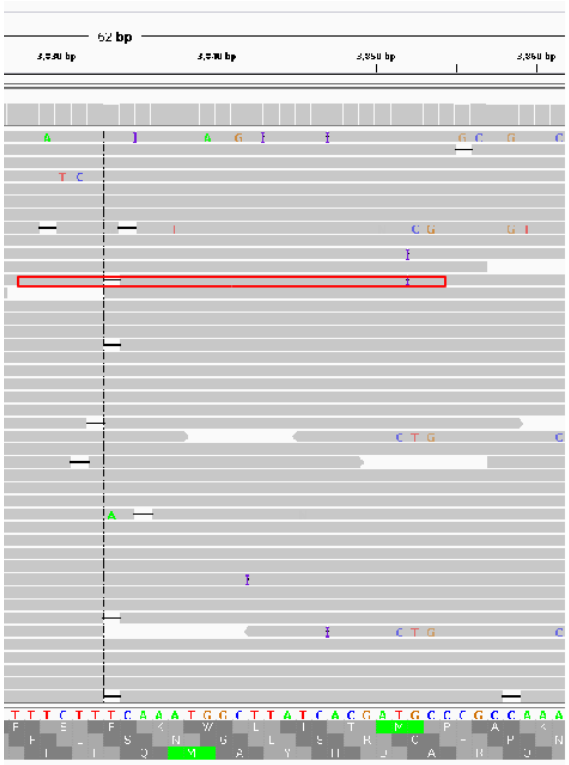


two downstream variants are
also enclosed in a red box

# settings

## (single read from 454)

- cutoff = 4.0 (x RMS error)
- number of molecules = 10,000
- simulation iterations = 1
- quality threshold = 40
- no drift – (window region -> no drift seen)
- alpha = 1E-9
- initial parameter values = 0.0005

# results

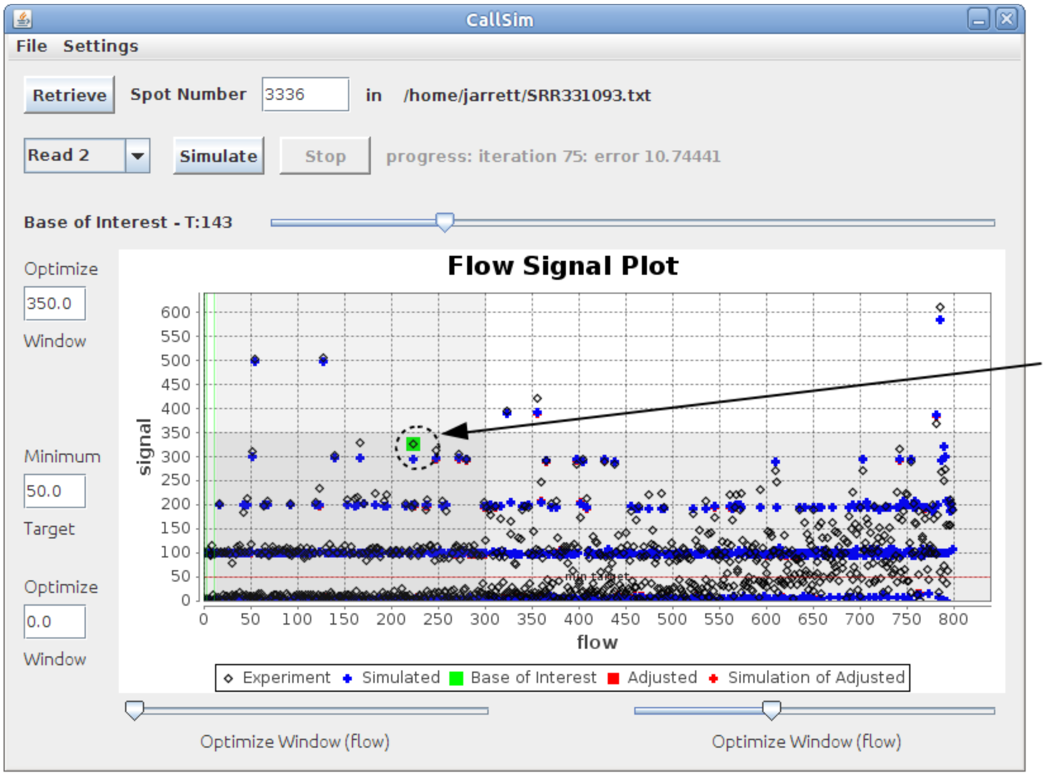


T variant @T143 supported

no other variants within the window

# alternate simulation settings

## (single read from 454)

- larger window – ends at later flow

- cutoff = 4.0 (x RMS error)
- number of molecules = 10,000
- simulation iterations = 1
- quality threshold = 40
- using drift – (some upward drift is seen in flow signal plot)
- alpha = 1E-8
- initial parameter values = 0.002

## results

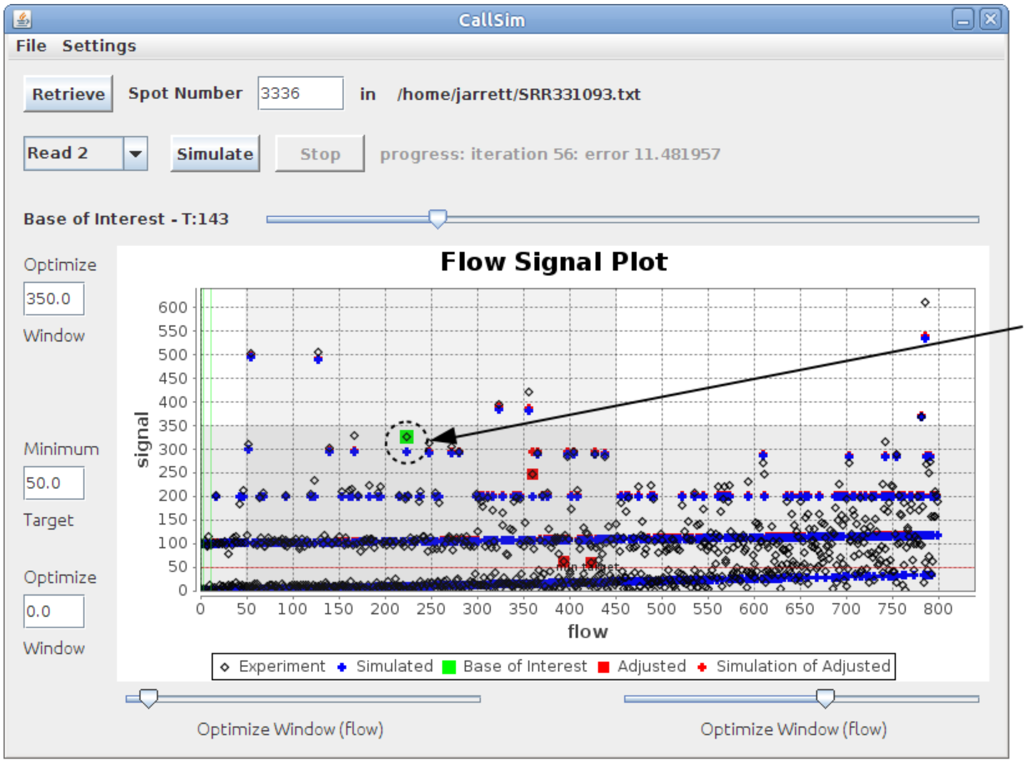


T variant @T143
supported

support for (red):

TT -> TTT @T235
eliminate gap

C -> _ @C254
eliminate insert

and
G -> _ @G273
elminate insert

# other locus of Interest – base 3591

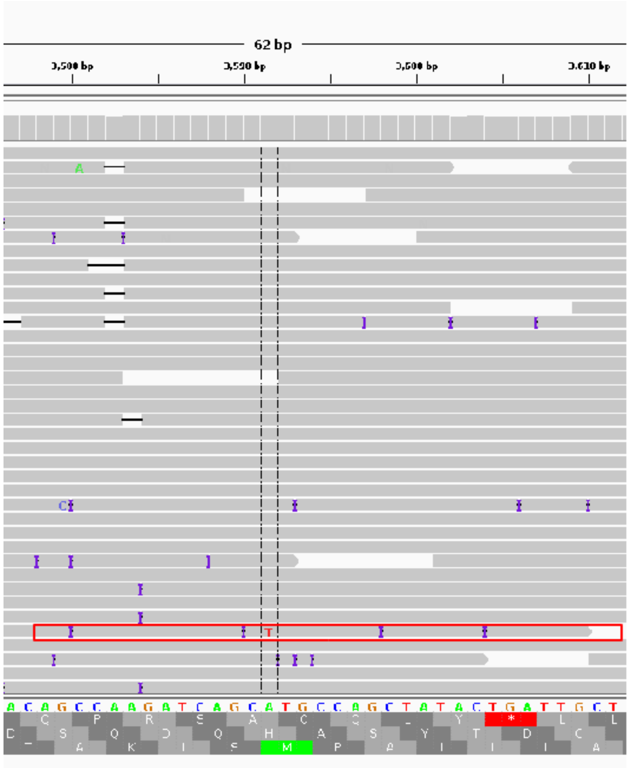


spot # 2335 outlined in red box

locus of interest from Macalalad et al.

rare variant listed as an error

has phred quality score of 16

# settings
## (single read from 454)

- cutoff = 4.0 (x RMS error)
- number of molecules = 10,000
- simulation iterations = 1
- quality threshold = 40
- no drift -
  - (upward drift less visible in homopolymer-call regions - 200 and higher signal levels)
- alpha = 1E-9
- initial parameter values = 0.0005

## results

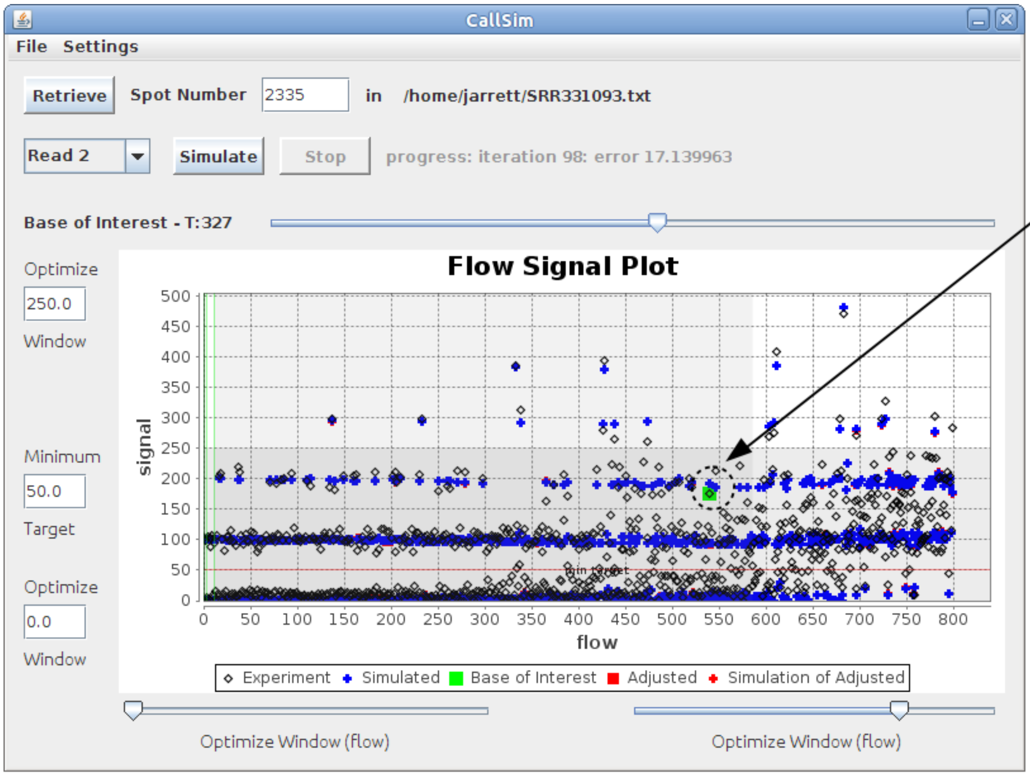


T variant @T327 supported

single base inserts seen in the mapping are not a focus, however with drift enabled, they can be evaluated better

# comments

Note: the adjustments can result in differences between “Simulated” and “Simulation of Adjusted” values in downstream flows because some extensions are carried forward.

CallSim analysis would follow the upstream processing steps necessary during post-processing of next-gen reads.

Rare variants can be evaluated by examining a small set of reads with CallSim.

# references

- MUMmer 3: http://mummer.sourceforge.net
- SRA Toolkit: http://www.ncbi.nlm.nih.gov/Traces/sra/?view=software
- Bowtie 2: http://bowtie-bio.sourceforge.net/bowtie2/index.shtml
- IGV: http://www.broadinstitute.org/igv/

**Validation Data**


- Mellmann et al., "Prospective Genomic Characterization of the German Enterohemorrhagic Escherichia coli O104:H4 Outbreak by Rapid Next Generation Sequencing Technology", PLoS One, 2011 July 20
- Rasko et al., "Origins of the E. coli Strain Causing an Outbreak of Hemolytic–Uremic Syndrome in Germany", NEJM, august 25, 2011


**Sequencing Technology**

- http://www.iontorrent.com/
- http://www.my454.com/
- sequence read archive: http://sra.dnanexus.com

**Test Data**


- Howden et al., “Evolution of Multidrug Resistance during Staphylococcus aureus Infection Involves Mutation of the Essential Two Component Regulator WalKR”, PLoS Pathog. 2011 November; 7(11)
- Macalalad et al., “Highly Sensitive and Specific Detection of Rare Variants in Mixed Viral Populations from Massively Parallel Sequence Data”, PLoS Comput Biol. 2012 March; 8(3)
